# AAV Vector-Mediated Antibody Delivery (A-MAD) in the Central Nervous System

**DOI:** 10.3389/fneur.2022.870799

**Published:** 2022-04-12

**Authors:** Marika Marino, Matthew G. Holt

**Affiliations:** ^1^Laboratory of Glia Biology, VIB-KU Leuven, Center for Brain & Disease Research, Leuven, Belgium; ^2^Department of Neurosciences, KU Leuven, Leuven, Belgium; ^3^Leuven Brain Institute, Leuven, Belgium; ^4^Synapse Biology Group, Instituto de Investigação e Inovação em Saúde (i3S), University of Porto, Porto, Portugal

**Keywords:** AAV vectors, AAV vector-mediated antibody delivery (A-MAD), Monoclonal antibodies, Nanobodies (VHH), Central Nervous System, Blood Brain Barrier (BBB)

## Abstract

In the last four decades, monoclonal antibodies and their derivatives have emerged as a powerful class of therapeutics, largely due to their exquisite targeting specificity. Several clinical areas, most notably oncology and autoimmune disorders, have seen the successful introduction of monoclonal-based therapeutics. However, their adoption for treatment of Central Nervous System diseases has been comparatively slow, largely due to issues of efficient delivery resulting from limited permeability of the Blood Brain Barrier. Nevertheless, CNS diseases are becoming increasingly prevalent as societies age, accounting for ~6.5 million fatalities worldwide per year. Therefore, harnessing the full therapeutic potential of monoclonal antibodies (and their derivatives) in this clinical area has become a priority. Adeno-associated virus-based vectors (AAVs) are a potential solution to this problem. Preclinical studies have shown that AAV vector-mediated antibody delivery provides protection against a broad range of peripheral diseases, such as the human immunodeficiency virus (HIV), influenza and malaria. The parallel identification and optimization of AAV vector platforms which cross the Blood Brain Barrier with high efficiency, widely transducing the Central Nervous System and allowing high levels of local transgene production, has now opened a number of interesting scenarios for the development of AAV vector-mediated antibody delivery strategies to target Central Nervous System proteinopathies.

## Introduction

**Figure 1:**
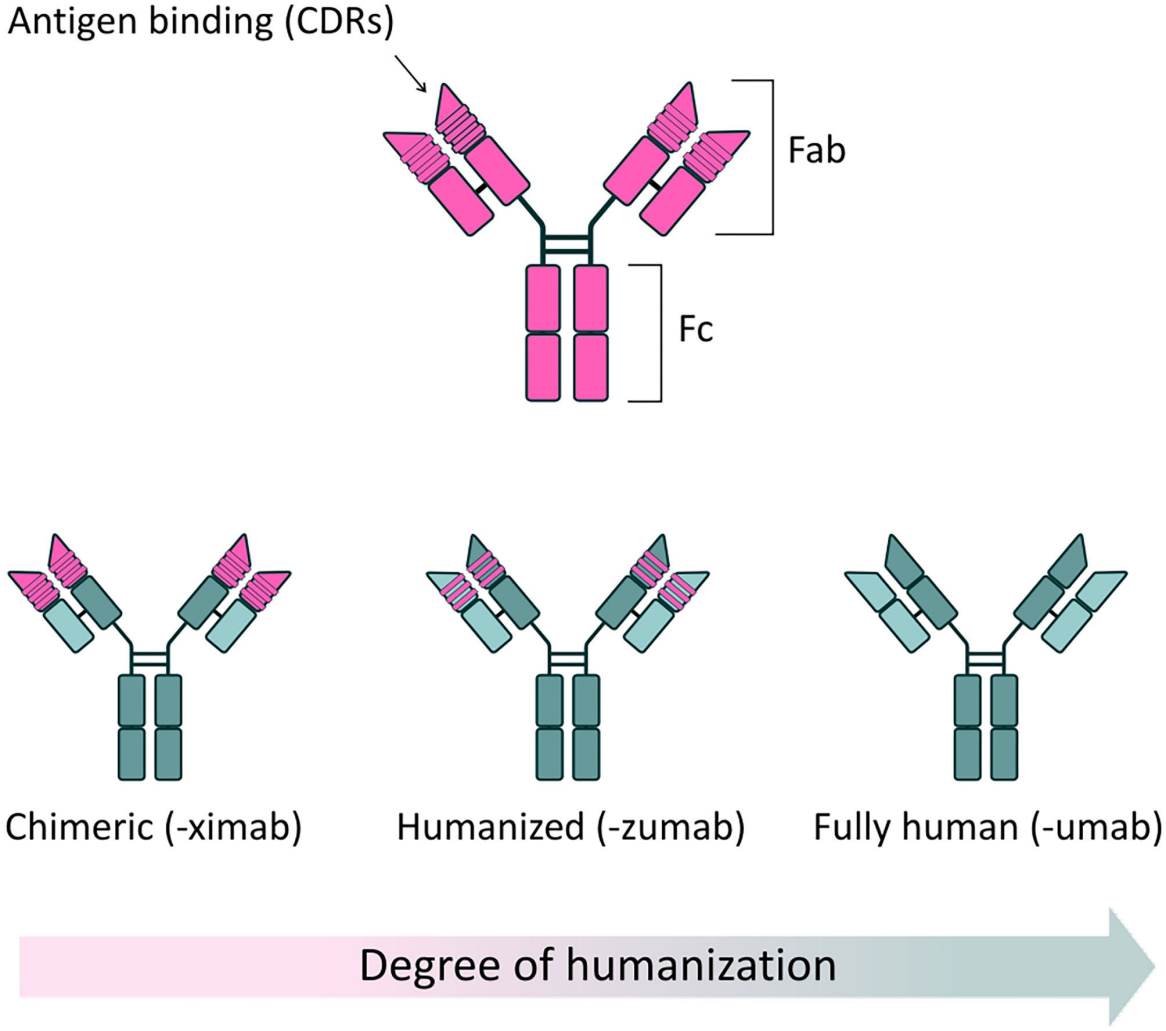
Full-length antibody structure. **(Top)** Schematic of a typical antibody structure, generated from a murine source. Two heavy chains and two light chains are connected in pairs by disulphide bonds. The fragment antigen binding (Fab) region contains highly variable complementarity determining regions (CDRs), responsible for the specific recognition and binding of antigens. The fragment crystallizable (Fc) region binds to specific receptors on immune cells, modulating the immune response, as well as some proteins of the complement system. **(Bottom)** Reduced immunogenicity of mAbs through protein engineering. The clinical success of mAbs has largely been driven by application of protein engineering techniques, which have allowed the development of safer, less immunogenic structures, when compared to mAbs of solely murine origin. Chimeric antibodies are characterized by approximately one third of their structure being derived from the original murine source. In these antibodies, murine-derived variable regions are linked to a human constant domain scaffold. By convention, chimeric mAbs are named using the “-ximab” suffix. In humanized mAbs, 90% of the structure is of human origin, with only the antigen binding sites deriving from the original mouse source. Humanized mAbs are named using the “-zumab” suffix. Fully human mAbs are entirely derived from human sources and thus display minimal immunogenicity in clinical use. Fully human antibodies are named using the “-umab” suffix.

Antibodies, or Immunoglobulins (Ig), are glycoproteins produced by the immune system, characterized by their ability to recognize and bind a specific region (epitope) of an antigen with high specificity and (generally) high affinity, neutralizing potential pathogens. The basic structure of an Ig, determined by X-ray crystallography, is a tetramer of ~150 kDa, formed by two identical pairs of heavy (50 kDa) and light polypeptide chains (25 kDa), joined by disulfide bonds. Heavy and light chains present highly variable complementarity determining regions (CDRs), which form the Fragment antigen binding (Fab) regions of the antibody, responsible for antigen recognition and binding. In contrast, the Fragment crystallizable (Fc) region binds to a variety of receptors on immune cells, to mediate antibody interaction with other components of the immune system and provide effector function (Figure 1).

The use of antibodies as therapeutic agents has increased considerably in the last 40 years, thanks to the generation of monoclonal antibodies (mAbs)—monovalent, laboratory-produced antibodies, derived from a single B-lymphocyte cell clone (1). Since the approval of the first therapeutic mAb, Muromonab-CD3 (OKT-3), by the US Food and Drug Administration (FDA) in 1985 (2), a further 80 mAb-based therapeutics have been approved for a range of clinical applications, with oncology, immunology and hematology being the predominant therapeutic areas (3, 4). This increased use of mAbs is no doubt partly fueled by progressive improvements in general safety, due to the development of less immunogenic chimeric (5, 6), humanized (7), and fully human structures (8) (Figure 1). mAbs have now achieved the status of best-selling pharmaceutical products: as of 2018, eight out of the ten best-selling drugs globally were mAbs; and in 2020 one single mAb, the anti-TNFα monoclonal Adalimumab (Humira®), generated an annual revenue of $19.9 billion (9). Interestingly, as of 5 years ago, ~570 mAbs were in evaluation in different phases of clinical trials and, based on previous completion rates, it is expected that a significant number of these novel mAbs therapeutics will come to market (10).

Proteinopathies are a large class of CNS diseases, characterized by the progressive accumulation of abnormal protein aggregates/inclusions, which are generally thought to confer a toxic gain-of-function (Table 1) (32). Generally, these toxic proteins accumulate intracellularly, although the toxic Aβ peptide classically associated with Alzheimer's disease accumulates extracellularly. Proteinopathies usually arise as adult onset, degenerative disorders, initially localized to specifically vulnerable regions of the CNS before spreading through the brain, possibly due to prion-like propagation mechanisms (33–36). Hence, drug delivery approaches that can widely target the CNS are required for the treatment of these severely debilitating conditions. In principle, mAbs represent an ideal therapeutic to treat these diseases, due to their high specificity for their protein targets [either directly targeting a toxic protein, or the processing enzymes responsible for its production, such as the β-site amyloid precursor protein cleaving enzyme 1 (BACE1) in Alzheimer's disease (AD)]. Importantly, the exquisite specificity of mAbs can be harnessed to target desired domains/functions of a protein while leaving the activity of other key domains intact (37). It can also be used to discriminate toxic protein variants from non-toxic forms, as shown by the ability of mAbs to distinguish different α-synuclein strains responsible for distinct synucleinopathies (38–40) and different structural variants of Aβ_1−42_ in Alzheimer's disease (AD) (41). This degree of targeting precision is superior to that achievable using alternative strategies, such as RNA interference (RNAi) and antisense oligonucleotides (ASOs), which lead to complete loss of protein function. RNAi and ASOs have also been reported to have additional problems linked to increased risks of toxicity (42–44) and off-target effects (45–47), and also appear unsuitable for depletion of long-lived proteins, as they operate at the transcriptional level, and so are dependent on protein depletion to have an effect (48).

**Table 1:** List of A-MAD approaches in preclinical models of the most common CNS proteinopathies.

**Protein**	**Proteinopathy**	**Antibody format**	**Delivery platform**	**Model**	**Reference**
α-synuclein^*^	Parkinson's disease and dementia with lewy bodies	VHH	rAAV5/ICV	Sprague–Dawley rats	Chatterjee et al. (11)
α-synuclein^*^		scFv	rAAV1/ICV	DAT-Cre rats	Chen et al. (12)
Tau^*^	Frontotemporal dementia	Full-length	rAAVrh.10/ICV	Tau P301S mice	Liu et al. (13)
Aβ^*^	Alzheimer's disease	scFv	rAAV1/ICV	3xTg-AD mice	Ryan et al. (14)
Aβ^*^		scFv	rAAV1/ICV	TgCRND8 mice	Levites et al. (15)
Aβ^*^		scFv	rAAV2/ICV	Tg2576 mice	Fukuchi et al. (16)
Aβ^*^		scFv	rAAV/ICV	3xTg-AD mice	Sudol et al. (17)
Aβ^*^		scFv	rAAV5/ICV	APPswe/PS1dE9 mice	Kou et al. (18)
Aβ^*^		Full-length	rAAV1/IM	Tg2576 mice	Shimada et al. (19)
Aβ^*^		scFv	rAAV1/IM	APPswe/PS1dE9 mice	Yang et al. (20)
Aβ^*^		scFv-IgG^**^	rAAV1/ICV	APP/PS1 mice	Elmer et al. (21)
Tau^*^		Full-length	rAAVrh.10/ICV	Tau P301S mice	Allen et al. (22)
Tau^*^		scFv	rAAV8/ICV	Tau P301S mice	Ising et al. (23)
Tau^*^		scFv	rAAV5/ICV	JNPL3 mice	Vitale et al. (24)
Tau^*^		scFv	rAAV1/ICV	rTg4510 mice	Goodwin et al. (25)
TDP-43^*^	Amyotrophic lateral sclerosis	scFv	rAAV9/IT	TDP-43^A315T^ mice	Pozzi et al. (26)
SOD-1^*^		scFv	rAAV1/IT	SOD1^G93A^ mice	Patel et al. (27)
SOD-1^*^		scFv	rAAV9/IV	SOD1^G93A^ mice	Ghadge et al. (28)
*PrP^*C*^*	Prion disease	scFv	rAAV2/ICV	TSE mice	Wuertzer et al. (29)
Huntingtin^*^	Huntington's disease	scFv	rAAV1/ICV	B6.HDR6/1 mice	Snyder-Keller et al. (30)
Huntingtin^*^		scFv	rAAV6/ICV	R6/2_Tg mice	Amaro and Henderson (31)

* *Evidence for prion-like cell to cell transmission*.

** *Engineered for Fc effector function*.

*ICV, Intracerebroventricular; IM, Intramuscular; IT, Intrathecal; IV, Intravenous; TDP-43, Transactive response DNA binding protein-43; SOD1, Superoxide dismutase 1*.

The use of mAbs as CNS therapeutics, however, is complicated by two main issues: (i) their large multimeric structure, limiting their ability to cross the Blood Brain Barrier (BBB) following intravenous administration (49) and (ii) their moderate half-life (~11–30 days in humans) (49). Combined, these issues mean that mAbs require repeated administration at high doses to maintain therapeutically relevant levels in the CNS, as shown by ^ch^Aducanumab (Aduhelm®), which requires monthly intravenous infusions to achieve disease modifying effects in AD (50). Repeated administration does, however, have a number of disadvantages. First, it can negatively impact on patient compliance. Second, it will result in accumulating costs, particularly for long-term, adult-onset neurodegenerative diseases, which will require high levels of dosing over a sustained period.

As such, the vast majority of mAbs currently on the market are indicated for the treatment of acute peripheral conditions, with oncology, autoimmune diseases and dermatology being the most prevalent therapeutic areas. In contrast, the development of mAbs therapeutics for the treatment of CNS disorders is lagging behind, albeit with a few exceptions, such as anti-CGRP and anti-CGRP receptor mAbs for the treatment of migraine (forecast to become best-selling drugs by 2024) (51), and the aforementioned ^ch^Aducanumab, regardless of the ongoing controversy surrounding its clinical efficacy.

Hence, to fully exploit the unique specificity properties of mAbs (and their derivatives) within the CNS, we believe it is essential to employ technologies that allow efficient BBB crossing and provide long-term, stable drug exposure in the CNS. In this respect, recombinant vectors based on Adeno-Associated Viruses (rAAV) have been identified as promising tools to unlock the potential of mAbs (and their derivatives) for the treatment of CNS proteinopathies. In this review, we will summarize the current status of the field and highlight areas of potential improvement that we believe will turn the prospect of rAAV vector-mediated antibody delivery (A-MAD) for the treatment of CNS proteinopathies into a reality in the near future.

## The Blood Brain Barrier

**Figure 2:**
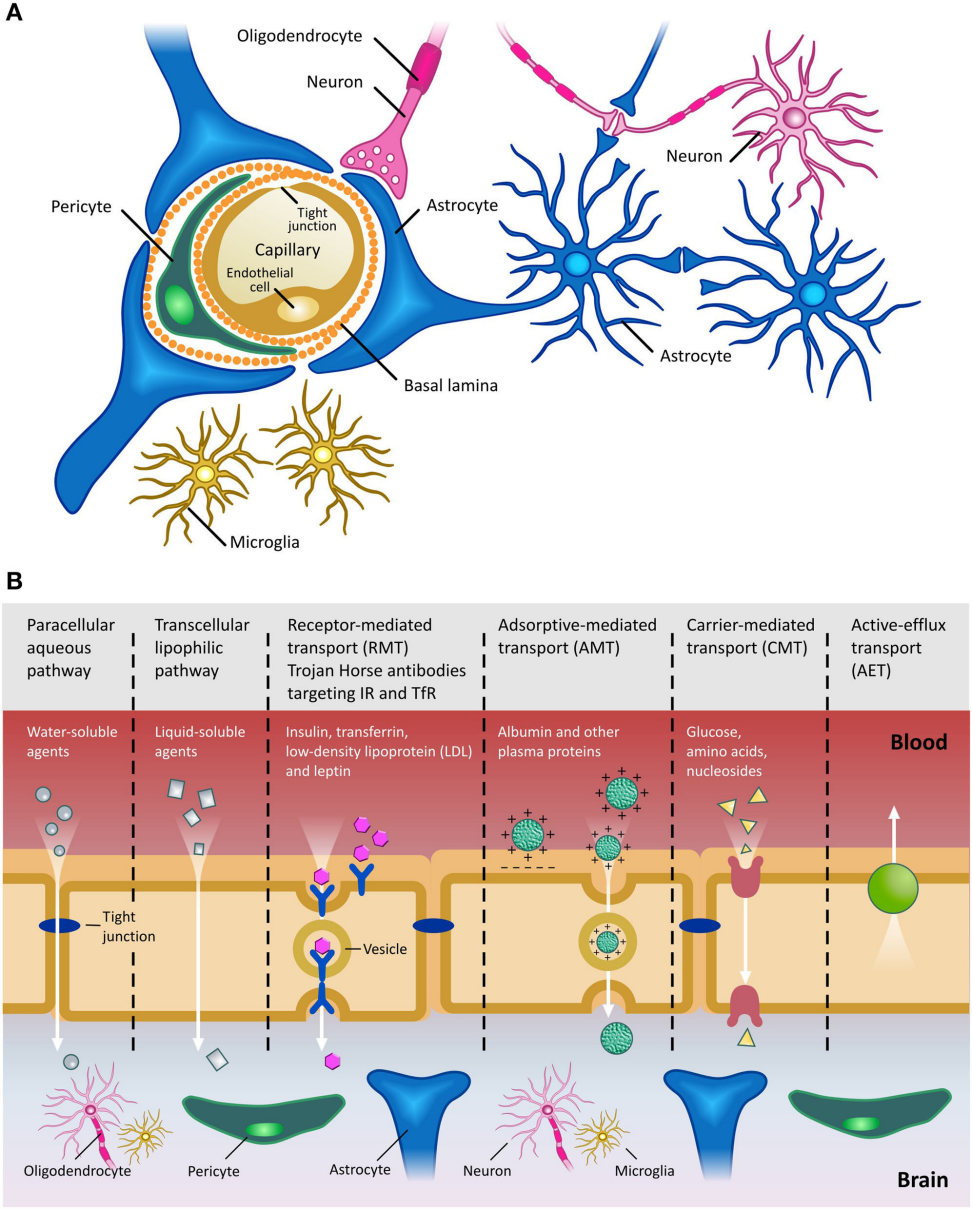
The Blood Brain Barrier. **(A)** Schematic of the BBB shown in cross-section. Main cellular components are shown. Endothelial cells are fused together by tight junctions, and surrounded by pericytes and astrocytic end feet. Figure adapted from (59). **(B)** Transport pathways across the Blood Brain Barrier. Ions, water and other small molecules necessary to maintain CNS homeostasis (such as glucose for metabolic support) can cross the BBB using a variety of mechanisms, including those based on passive diffusion, from high to low concentrations, and active transport, requiring ATP hydrolysis. Molecules can cross the BBB using either paracellular (between cells) or transcellular (across cells) pathways. Receptor-mediated transcytosis (RMT) allows selective uptake of macromolecules, including plasma proteins, enzymes, hormones and growth factors, via receptors expressed on the luminal side of the BBB. Trojan horse antibody strategies exploit RMT to enable delivery of conjugated drugs within the CNS. Adsorptive-mediated transport (AMT) relies on the electrostatic interaction between positively charged molecules (in general charged peptide moieties) and the negatively charged plasma membrane. Carrier-mediated transport (CMT) is an energy-dependent mechanism, allowing co-transport of molecules, either in the same direction (symport) or in the opposite direction (antiport). Active efflux transport (AET) is involved in the clearance of molecules, including drugs, from the brain. Due to the general impermeability of the BBB and the reliance on specialized and specific transport mechanisms for transport of molecules into the CNS, high molecular weight therapeutics, such as mAbs and their derivatives, are largely excluded from the CNS. Figure adapted from (60).

“Blood brain barrier” is a working terminology that defines the microvasculature of the CNS, which possesses a unique structure and properties [extensively reviewed in (52)] (Figure 2A). The BBB allows the selective transport of molecules essential to maintain the CNS microenvironment and proper CNS function, while acting as a barrier against toxic compounds and dangerous microorganisms (Figure 2B). The shielding effect of the BBB, however, also severely limits the efficient penetration of therapeutics from the periphery into the CNS. In general, compounds which have a molecular weight >400 Da and are capable of forming more than 8 hydrogen bonds are efficiently excluded from the CNS by the BBB (53). This is especially problematic in the case of antibody-based therapies, due to their size. Typically, only 0.1–0.2% of systemically injected antibody accesses the CNS. Clearly, if therapeutic mAbs are not accessible to the brain, their utility for the treatment of proteinopathies will be limited. Hence, given the benefits of antibody-based therapies and the increasing unmet clinical need presented by CNS diseases, there is an ongoing search for technologies which can circumvent the CNS and efficiently deliver antibody-based therapeutics into the parenchyma (54) (see Section ‘Delivery of mAbs and their derivatives to the Central Nervous System’ below).

Disruption of the BBB has been reported at late stages of several proteinopathies, such as AD, Parkinson's disease (PD), Huntington's disease (HD) and Amyotrophic lateral sclerosis (ALS) (55, 56). In these conditions, in fact, multiple neurodegeneration pathways are driven by immune responses (associated with the influx of toxic blood-derived debris, cells and pathogenic microorganisms), as well as impaired oxygen transport and consequent hypoxia associated inflammation (56, 57). However, it is unclear whether such disruption would facilitate the entry of therapeutics into the CNS and, if it did, whether any benefits would offset the deleterious effects of barrier breakdown. Furthermore, the degree of BBB breakdown is likely variable and incomplete, suggesting that the use of BBB-crossing technologies to deliver antibodies into the CNS would still allow higher levels of therapeutic to be achieved (58), as shown in preclinical models of neurodegenerative disease with various degrees of BBB disruption. Finally, postponing drug administration until an advanced stage of disease, when extensive BBB disruption is potentially observed, would in all likelihood limit the therapeutic benefits, since significant (and currently irreparable) CNS damage would already have occurred. As such, developing novel technologies to circumvent the intact BBB, allowing the efficient delivery of mAbs into the CNS, is essential if we are to harness their full therapeutic potential for the treatment of CNS proteinopathies.

### Delivery of mAbs and Derivatives to the Central Nervous System

One way to overcome the shielding effect of the BBB is to inject therapeutics directly into the brain parenchyma. However, not only are direct injections highly invasive and damaging to the local tissue, but they are typically characterized by limited distribution of the therapeutic, which is retained near the injection site, limiting the use of this approach to treat multi-region CNS diseases (61–65). To some extent, these problems can be overcome by direct injection into the cerebrospinal fluid (CSF), but this method is also associated with potentially severe adverse effects, particularly if large boluses of drugs are rapidly administered (66). Drug entry into the CNS can also be achieved using administration of hyperosmotic solution (67) or by use of ultrasound to temporarily disrupt the BBB (68), although random opening of the barrier for undefined periods of time also involves the risk of potentially severe adverse effects (56). Ideally, the treatment of multi-region CNS diseases requires a drug delivery system which combines minimal invasiveness with widespread drug delivery.

To date, the most commonly exploited technology for the minimally invasive delivery of drugs to the CNS has been based on the naturally occurring process of receptor-mediated transcytosis (RMT), which serves to shuttle essential metabolites to the brain. This process can be subverted by so-called “Trojan Horses,” mAbs against endogenous BBB receptors, such as the insulin receptor (IR) or transferrin receptor (TfR). These antibodies bind their targets and are effectively carried across the BBB by RMT. By fusing the biologic of interest to the “Trojan Horse,” it is possible to obtain CNS delivery, following a single systemic bolus (69). This approach also underlies alternative technologies, such as the so-called “Brain Shuttle” (BS), which utilizes an antibody fragment against the TfR to transport biologics into the CNS (70). However, the “Trojan Horse” strategy is characterized by limited efficiency (CNS uptake of 1.1% and 3.5% of a systemically injected dose of TfR- or IR-targeting mAb in rhesus monkey, respectively) (71) and suffers from safety concerns linked to chronic administration of high antibody doses (71, 72). The widespread adoption of RMT-based technologies is impacted by a number of further issues. These include the fact that insulin and transferrin receptors are not uniquely expressed at the BBB site, raising safety concerns linked to non-site-specific distribution of the therapeutic (73). Potential solutions to overcome these concerns are currently being investigated, as shown by reports suggesting that a reduction in the antibody binding affinity to the TfR increases brain uptake, while mitigating adverse effects observed in the periphery (e.g., reduction in reticulocyte count) (73, 74). Additionally, receptor-specific “Trojan Horse” antibodies, such as anti-IR or -TfR are generally species-specific, requiring tuning of the binding profile in each species tested, which will negatively impact on the ease of their translation into humans (75, 76).

In contrast, rAAV vectors are emerging as ideal candidates for mAbs delivery to the CNS, as described below.

## rAAV Vectors for *in vivo* Gene Transfer to the CNS

**Figure 3:**
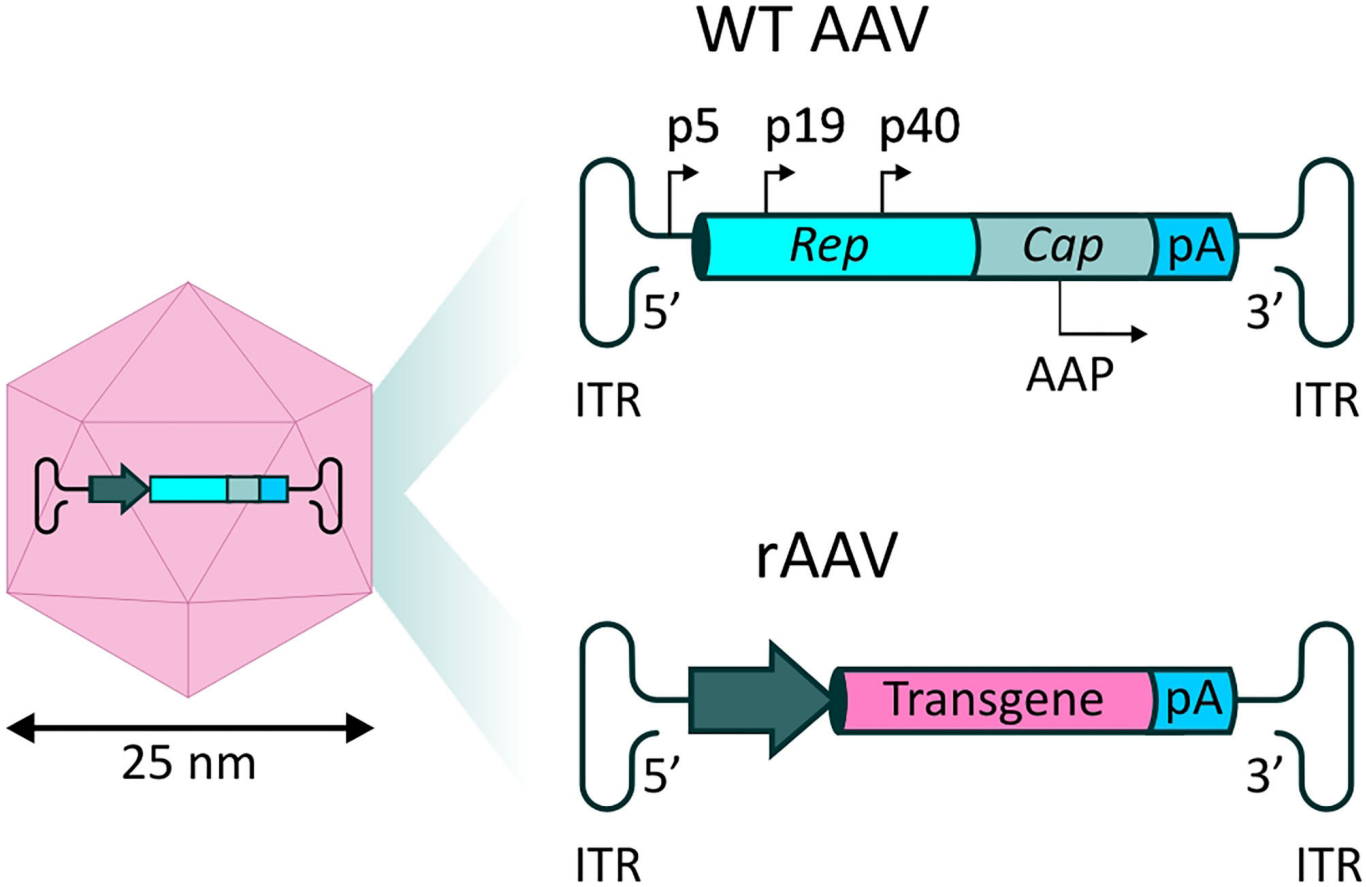
rAAV vectors for systemic delivery of mAbs. **(Left)** AAV capsid structure. The AAV capsid is composed of 60 protein monomers (VP1, VP2, and VP3 subunits with a stoichiometry of 1:1:10) arranged in icosahedral symmetry. **(Top right)** Representation of the single stranded (ss) DNA genome found in naturally occurring AAVs. Two open reading frames (ORFs) express genes necessary for replication (*rep*) and capsid structure (*cap*). An alternate reading frame overlapping the *cap* gene encodes the assembly-activating protein (AAP), promoting capsid formation and stability. **(Bottom right)** In recombinant AAV-based vectors (rAAV), the viral genes are substituted by a synthetic transgene cassette, in which a promoter drives expression of a transgene of interest, followed by a polyA tail.

Naturally occurring AAV is a non-enveloped virus, in which a single stranded (ss) DNA genome of ~4.8 kb is contained within an icosahedral protein capsid. The genome encodes three genes which act in various processes relating to the viral life-cycle. The four proteins encoded by the *rep* gene (Rep78, Rep68, Rep52, and Rep40) are required for viral genome replication and packaging, while expression of the *cap* gene produces viral capsid proteins (VP1, VP2, and VP3 subunits). Finally, an alternate reading frame overlapping the *cap* gene encodes the assembly-activating protein (AAP), which promotes interaction between VPs for capsid formation and stability. These coding sequences are packaged between two inverted terminal repeats (ITRs), essential for genome replication and packaging (77) (Figure 3). The initial interaction of AAV with cells, prior to infection, is mediated by interactions with specific carbohydrates on the surface of target cells, such as sialic acid, galactose and heparin sulfate (78). The preferential binding for one or more of these molecules, determined by differences in the sequences of the different VP proteins, determines the tropism of specific AAV serotypes.

Recombinant AAV (rAAV) vectors are formed by substitution of the viral genes with a synthetic expression cassette, in which a promoter element drives the expression of the therapeutic transgene of interest, followed by a polyadenylation (polyA) signal (Figure 3). In the typical rAAV vector production method, *rep* and *cap* are supplied *in trans*, alongside a helper plasmid containing essential genes (E4, E2a, and VA) which play a role in AAV replication (79). Standard ss rAAV have an optimal transgene capacity of ~4.8 kb, including regulatory sequences, such as promoters and regulatory elements, including the widely used polyadenylation sequence (polyA tail); packaging of coding sequences exceeding 5.2 kb is inefficient, and unfavorably impacts on transduction (80).

Over the past 30 years, rAAV vectors have been employed in a variety of clinical trials, both for peripheral (NCT03588299, NCT03001830, NCT02396342 for the treatment of hemophilia A and B) and CNS conditions (NCT01621581, NCT01973543, NCT03562494 for the treatment of Parkinson's disease; NCT00087789, NCT03634007 for the treatment of Alzheimer's disease) (81). These trials have shown rAAVs to have a largely safe clinical profile. One area of concern with systemic administration of high vector doses is potential hepatotoxicity, which has been reported in some clinical trials. However, it appears that this can be managed using corticosteroid administration (82). The FDA and the EMA approval of Zolgensma^®^, a pioneering AAV9-based medication for the treatment of Spinal Muscular Atrophy (SMA) in pediatric patients, is potentially the tip of the iceberg for AAV use in treating CNS diseases. In fact, as of January 2022, ~50 additional clinical studies employing rAAV vectors for the treatment of CNS disorders had been either completed or were ongoing (www.clinicaltrials.gov) (83).

### rAAV Vector-Mediated Antibody Delivery (A-MAD)

Convincing proof-of-principle has been obtained for rAAV vector-mediated for the treatment of peripheral conditions. In these studies, AAV vectors have been employed as gene transfer vehicles to deliver the coding sequence for specific antibodies into non-hematopoietic cells, driving antibody production and secretion for pre- or post-challenge treatment (84). This approach, known as Vectored Immunoprophylaxis (VIP), induced lifelong expression of significant doses of mAbs in multiple models of disease for the prevention of infection with various pathogens (85), such as Influenza A (86) and Hepatitis C (87) viruses, *Plasmodium falciparum* (88) and HIV (89–91). Such has been the success of this approach that VIP is currently being tested in phase 1 clinical trials (NCT01937455; NCT03374202) in HIV-infected adults (92).

Based on this accumulated weight of evidence, it is our opinion that the outstanding therapeutic potential of antibodies offers a unique opportunity to target CNS proteinopathies. Significant benefits of A-MAD strategies have emerged in preclinical models of CNS diseases (Table 1), and the translation of this technology into a therapeutic strategy for humans has now become a priority. We believe that combining mAbs derivatives and rAAV vectors truly has the potential to offer long-awaited therapeutic options against chronic and/or degenerative CNS proteinopathies, as (i) certain AAV serotypes have the ability to cross an intact BBB in multiple animal species, including non-human primates, resulting in widespread CNS transduction (93); (ii) rAAV vectors are able to infect the non-dividing (post-mitotic) cells of the CNS and are considered safe, due to their non-replicative and non-integrating nature; (iii) rAAV vectors provide stable, long-lasting production of the therapeutic of interest [up to 15 years in a non-human primate parkinsonian model (94)]; and (iv) due to the vector life-cycle, therapeutics are produced within the cell and, therefore, are able to access intracellular targets with ease (83).

### Systemic Delivery of AAV Vectors to Target CNS Disorders

Systemic administration of rAAV vectors with BBB crossing properties arguably represents the best option for treating multi-region CNS disease using mAbs (or derivatives). Unlike approaches relying on local vector administration, such as intraparenchymal (IP) (95), intrathecal (IT) (96, 97) and intracerebroventricular (ICV) (98) injections, in which transduction is largely limited to the area surrounding the injection site, systemic delivery results in widespread transduction of the CNS. However, systemic administration does require the use of much higher vector doses to achieve adequate levels of CNS transduction, raising concerns about potential immunotoxicity (99). Although systemic delivery of AAV vectors has proved to be largely safe in a number of clinical trials targeting peripheral conditions [e.g., Spinal Muscular Atrophy Type I (NCT02122952); Duchenne Muscular Dystrophy (NCT03368742); and Mucopolysaccharidosis (NCT03315182) (100)], potential concerns linked to off-target transduction of peripheral organs and immune responses to the AAV capsid and/or the transgene cannot be completely discounted, particularly when targeting adult CNS conditions, which will generally require higher rAAV vector doses than those administered to children.

**Figure 4:**
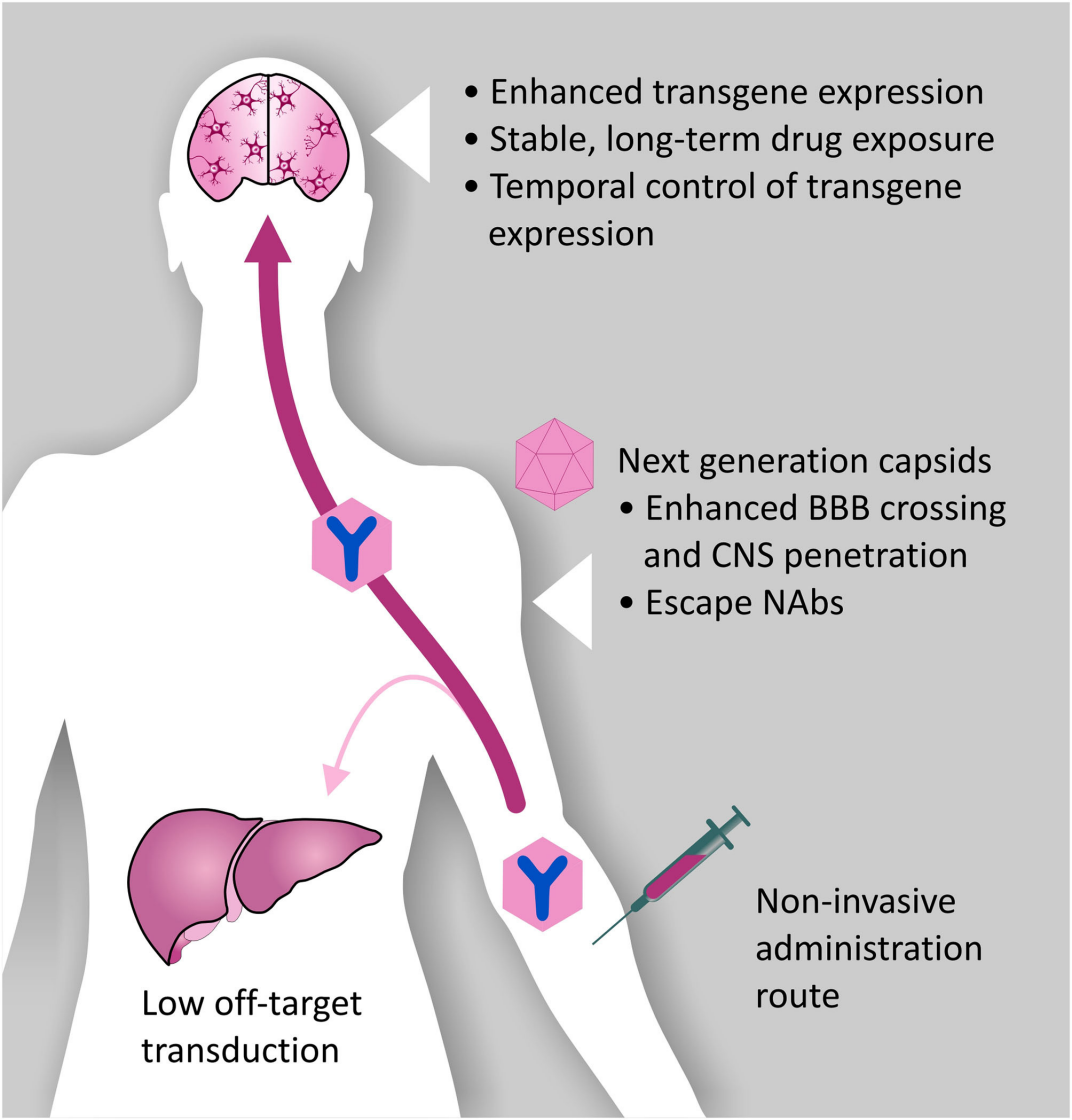
Desired characteristics of rAAV vectors for A-MAD. Certain rAAV vector serotypes show BBB crossing properties, thus allowing minimally invasive and long-term delivery of mAbs and their derivatives within the CNS. However, improvements to the rAAV vector platform are desirable to maximize the therapeutic potential of A-MAD strategies, and decrease the risk of potential side effects. In particular, capsid engineering can be used to enhance BBB penetration, and enable CNS cell-type specific tropism. These modifications would also have the benefit of reducing potential off-target toxicity which, in the case of systemic administration, is usually linked to liver transduction. Introduction of capsid modifications is also desirable to reduce the likelihood of vector capture by pre-existing neutralizing antibodies (NAbs), which have been found to severely limit the efficiency of the system. In parallel, engineering of the rAAV transgene cassette is desirable to maximize transgene expression efficiency, thus allowing high (therapeutically relevant) levels of mAbs (or derivatives) within the CNS. Moreover, following systemic administration, it will be essential to spatially restrict transgene expression, de-targeting the periphery. The ability to turn transgene expression “on” or “off” will serve to prevent, or limit, adverse reactions to the transgene. Incorporation of such modifications into basic vector design will, in all likelihood, allow remaining concerns over the safety of AAV systems for CNS use to be overcome, while also increasing their therapeutic potential.

Consequently, introduction of modifications to the rAAV vector capsid, responsible for vector tropism, and the transgene cassette, have become increasingly important to (i) minimalize safety concerns, limiting unwanted transduction of peripheral organs (ii) improve BBB crossing, and (iii) improve transgene expression levels in a cell-type specific manner. A major issue with systemic AAV administration is the presence of pre-existing neutralizing antibodies (NAbs) in approximately half of the human population, as a result of exposure to naturally occurring AAVs. NAbs are durable and display considerable cross-reactivity across serotypes, severely hindering transduction efficiency (101). Engineering the rAAV vector capsid to evade NAbs is, therefore, an important strategy to improve the efficiency of systemic delivery and A-MAD in the CNS (Figure 4). In the following sections, we will discuss recent innovations in the fields of capsid and transgene engineering, that we believe will help facilitate the use of A-MAD to treat CNS proteinopathies.

## Improvement of AAV Vectors for A-MAD: Engineering the Capsid

Important advances in the clinical application of rAAV vectors have mostly been based on AAV9, as this serotype is characterized by higher transduction efficiency in the CNS, cardiac and skeletal muscle, pancreatic tissue and liver in comparison with other serotypes (102), and lacks the proinflammatory cytokine induction typically observed with lentiviral vectors. The ability of AAV9 to cross the BBB from the peripheral circulation and transduce non-dividing cells in the CNS has been behind the successful development of Zolgensma, with several other therapeutics currently being investigated for the treatment of CNS proteinopathies. An additional advantage of AAV9 is that the prevalence of anti-AAV9 NAbs in humans is lower than that observed for other serotypes, such as AAV1 and 2 (103). Given the useful properties of this serotype, we believe that further engineering of the AAV9 capsid could generate highly efficient BBB crossing capsids for use as minimally invasive delivery platforms for mAbs (and derivatives) for the treatment of CNS disorders.

### Engineering the AAV Capsid Using Rational Design

Knowledge of AAV biology, including aspects of capsid structure, assembly and transduction mechanism, has allowed engineering of vectors with the aim of enhancing, or introducing, new and beneficial characteristics (104). This strategy is known as rational capsid design. Through rational design, novel AAV serotypes, showing enhanced specificity for CNS cell-types in comparison to peripheral organs, have been produced. Rational design generated AAV9.HR, a serotype able to preferentially transduce neurons upon systemic administration in neonatal mice, while de-targeting the periphery (105). AAV9.HR was derived from the parental serotype AAV9 by introducing the mutations His527Tyr and Arg533Ser, which influence galactose and LamR receptor binding (105, 106).

Rational capsid design has also allowed creation of entirely artificial AAV capsids, using phylogenic analyses to enable *de novo* gene synthesis of ancestral capsid proteins from the common ancestors of AAVs currently in circulation. These reconstructed ancestral capsids possess intriguing features, such as an enhanced thermostability and wide transduction patterns (107) that could open interesting scenarios for the delivery of mAbs (and derivatives) to a targeted CNS cell-type. Particularly relevant for CNS targeting is serotype Anc80L65, which has shown a 4-fold increase in the ability to transduce astrocytes, when compared to AAV9, following intravenous administration in adult mice (108). Preferential targeting of astrocytes via A-MAD could lead to significant benefits for the treatment of Transactive response DNA binding protein of 43 kDa (TDP-43)-related proteinopathies, such as ALS and frontotemporal dementia (FTD), where astrocyte activation and inflammation appear as strong effectors in disease progression (109). Of interest, a current gap in rAAV platform technology is the lack of serotypes able to efficiently transduce microglia, in our opinion one of the most valuable therapeutic targets in the CNS, due to their involvement in numerous proteinopathies (110–112). To date, modification of the AAV6 capsid through site-directed mutagenesis has been shown to increase transduction efficiency in monocyte-derived dendritic cells (113). However, microglia remain largely resistant to transduction (114). Further research efforts, directed toward the identification of microglia-targeting rAAVs, can open interesting scenarios for A-MAD strategies targeting the inflammatory component of CNS proteinopathies, although the potential impact of cell-transduction on the activation state of microglia may prove to be a significant challenge (115).

### Engineering the AAV Capsid Using Directed Evolution

Modification of AAV capsids can also be achieved through methods that do not rely on *a priori* information, but on random events [capsid shuffling (116), peptide insertion (117), random mutagenesis (118)]. These approaches, known collectively as directed capsid evolution methodologies, have succeeded in generating rAAV capsids with remarkable BBB crossing properties and cell-type specific tropism following systemic administration (119).

Capsid shuffling generates hybrid serotypes such as AAV-Olig001, which was created by fusing together elements of different capsids originating from AAV serotypes 1, 2, 6, 8, and 9. AAV-Olig001 shows exceptional specificity for oligodendrocytes (95%) after intravenous administration in rats, with no reported transgene expression in astrocytes and microglia (120).

To date, perhaps the most successful strategy for AAV capsid evolution aimed at improving BBB crossing has been through modification via peptide insertion. This methodology forms the basis of the Cre recombination-based AAV directed evolution (CREATE) platform (121). In this method, a library of capsid variants was generated by introduction of a randomized sequence of 7 amino acids (aa) between aa 588 and 589 of the VP1 capsid subunit from AAV9. The capsid library was engineered around an expression cassette encoding a Cre-inducible fluorescent reporter, allowing recovery of capsid variants which successfully transduced cells in a Cre-expressing mouse (astrocyte-specific *Gfap*-promoter) following systemic injection. CREATE was used to generate the serotypes AAV-PHP.B, AAV-PHP.eB and AAV-PHP.S, which show enhanced BBB penetration with concomitant increases in brain (121) and spinal cord transduction (122), in comparison to the parental serotype. Unfortunately, the increased BBB crossing seen with AAV-PHP.B appears limited to C57Bl/6J mice, with other mouse strains and animal species (including non-human primates) showing limited CNS transduction following systemic administration, due to a lack of the obligate LY6A receptor on endothelial cells of the brain vasculature (123, 124). This limitation could be potentially overcome by the work of Hanlon et al. (125), who described a peptide insertion strategy, iTransduce, in which pseudorandom 21-base nucleotides were inserted between amino acids 588–589 of the AAV9 VP1 capsid subunit. Selection of BBB crossing capsids in iTransduce was again based on Cre-inducible marker expression, albeit in this case Cre was encoded by the AAV expression cassette and the vector was systemically applied to a fluorescent reporter mouse. This strategy was used to generate AAV-F, with a transgene expression efficiency in the murine brain comparable to that of AAV-PHP.B following intravenous administration. However, AAV-F does not rely on the LY6A receptor for BBB crossing, and it appears able to efficiently cross the BBB in non-C57Bl/6J genetic backgrounds (BALB/c) after systemic administration. To date, however, it is impossible to foresee whether AAV-F could be a clinically useful serotype, as it has not been systemically administered in non-human primates. In all likelihood, ongoing research efforts will provide us with a plethora of novel BBB crossing rAAV serotypes, allowing efficient targeting of CNS disorders in humans.

As introduced earlier, capsid modifications are also, to date, the most successful aid to AAV vector evasion of both capsid specific cytotoxic T-lymphocytes (CTL) and innate immune responses upon vector administration (126). For example, introduction of mutations in the epitope on the AAV2.5 capsid recognized by the monoclonal antibody A20 reduces levels of vector neutralization (127); while the chimera AAV-DJ shows the ability to evade neutralizing antibodies generated by exposure to other serotypes (128). In our opinion, the benefits of engineering new, immunosilent AAV serotypes exceed those of alternative strategies required to maximize the efficiency of systemic AAV administration and its use in A-MAD. Administration of empty “decoy” capsids, use of immunosuppressants in patients (129) and plasmapheresis have all been proposed as useful strategies but, in fact, all pose some concerns. To begin with, as NAbs recognize the same epitopes on both empty capsids and those carrying the therapeutic transgene, extremely high doses of “decoy” capsids (~10-fold higher) are needed to ensure that NAbs are effectively mopped up, leaving the therapeutic vector free to cross the BBB and transduce the CNS. In contrast, immunosuppressive agents and plasmapheresis are non-specific and effectively remove all circulating antibodies, leaving patients exposed to the risk of opportunistic infections (130). To overcome this issue, plasmapheresis methods based on AAV-specific immune absorption columns are being tested for their ability to exclusively deplete anti-AAV antibodies in non-human primates; the safety and efficacy of such an approach is yet to be determined in humans (131).

## Improvement of AAV Vectors for A-MAD: Optimizing the Transgene Cassette

Upon successful cell transduction, a critical issue for successful A-MAD is the production and maintenance of a therapeutic threshold of biologics within the CNS. Production of the therapeutic transgene depends on conversion of the rAAV vector single stranded genome into double-stranded DNA (dsDNA), to enable subsequent mRNA transcription and translation. Synthesis of the second strand of DNA is considered a rate limiting step, delaying the onset of transgene expression and, in cells with particularly inefficient second strand synthesis, potentially limiting the steady-state level of therapeutic that can be produced (132, 133). This issue can be effectively overcome by creating a synthetic double-stranded genome, through mutating one of the ITRs so that it can form a short DNA hairpin—the so-called self-complementary (sc) configuration (134, 135). The sc configuration does, however, involve a 50% reduction in the transgene cassette capacity (~2.4 kb), thus limiting the choice of potential transgene candidates (136). As the cDNA of full-length mAbs occupies a minimum 2 kb of packaging space, A-MAD strategies using full length antibodies tend to accommodate a single copy of the antibody coding sequence in a single stranded genome (137, 138), which also includes standard regulatory elements, such as promoter sequences, the polyA signal and the woodchuck hepatitis virus post-transcriptional regulatory element (WPRE, see Section ‘Enhancing VHH Expression’ below). On the contrary, mAbs derivatives, due to their small size, are optimal for incorporation into a scAAV system.

### Derivatives of Monoclonal Antibodies for A-MAD

Insertion of full-length mAbs within a rAAV expression cassette often requires extensive reconfiguration, which can potentially alter antibody properties. mAbs derivatives of smaller size are therefore better suited for rAAV vector-mediated expression (139). In particular, camelid derived nanobodies, also known as VHH, are emerging as ideal candidates for incorporation into rAAV vectors, due to their single binding domain, exceptional stability and lack of an Fc effector function. This latter characteristic could contribute to a more favorable pharmacokinetic profile within the CNS, in comparison with full-length mAbs. In fact, it has been hypothesized that the binding of the Fc domain to Fc receptors in the BBB represents a potential route for the reverse transcytosis of antibodies across the BBB, facilitating their clearance from the brain (Figure 2B) (140–142).

**Figure 5:**
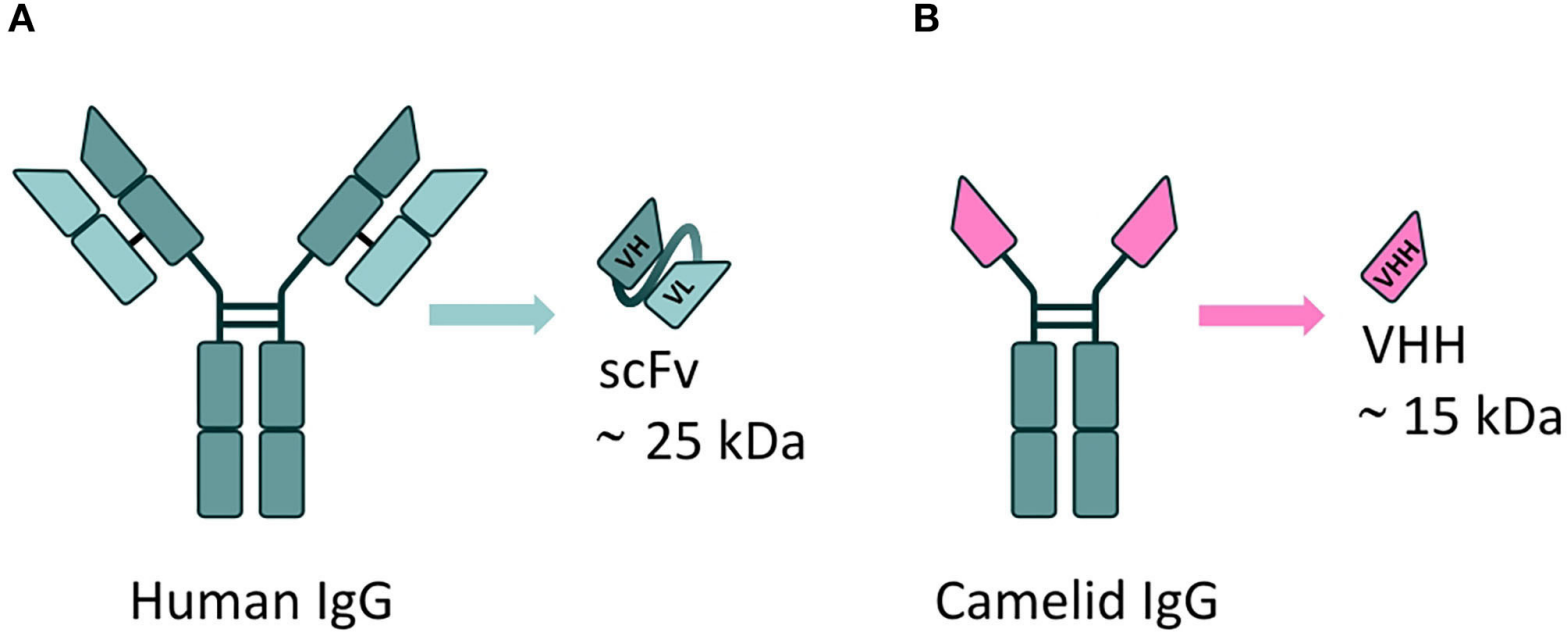
mAbs derivatives for A-MAD. **(A)** Representative scheme of a single-chain fragment variable (scFv) antibody fragment, derived from a human immunoglobulin. This class of engineered antibody is composed of the variable regions of the heavy (VH) and light chains (VL) fused by a linker. Their applicability is limited by potential folding and stability issues in the reducing environment of the cell. **(B)** Representative scheme of a VHH, the smallest antigen-binding functional fragment in nature, derived from a camelid immunoglobulin. The small size, high antigen specificity and exceptional stability of VHH (including in the reducing environment of the cell) make them an ideal cargo for rAAV vector-mediated delivery to target CNS proteinopathies.

VHH are the smallest naturally derived antigen-binding functional fragments (~15 kDa) (143), and as such are able to target epitopes inaccessible to full-length mAbs, such as the active site of enzymes (to modulate their catalytic activity) (144). The use of VHH largely overcomes the issues associated with the use of the single-chain fragment variable (scFv) format; to date, this is the most commonly employed antibody derivative used in A-MAD. However, scFv are notoriously difficult to engineer; scFv are formed from the variable regions of antibody heavy and light chains, joined by a short peptide linker, which requires extensive optimization to ensure correct protein folding and efficient antigen binding (145, 146), and they are frequently unstable in the highly reducing environment of the cell (147) (Figure 5).

One major advantage of the small size of VHH is the possibility to engineer multiple copies within a standard (sc) AAV expression cassette. This capacity can be used to introduce multiple copies of the same VHH to boost overall production levels, or to incorporate VHH raised against different targets. These could be targeting different domains within the same protein, to increase avidity, or completely independent proteins within a common signaling pathway. Employing such a strategy would significantly enhance the therapeutic potential of A-MAD in treating CNS proteinopathies, in a manner similar to that resulting from the use of multi-specific antibody fragments [bispecific T cell engagers (BiTEs) (148), diabodies (149) and dual affinity re-targeting antibodies (DARTs) (150)] for the treatment of peripheral conditions. Interestingly, VHH can be further engineered without loss of functionality, to (i) direct them into specific trafficking pathways and improve target engagement (151, 152), (ii) incorporate specific proteolysis-promoting sequences (PROTACs), to stimulate intracellular degradation of (toxic) proteins involved in CNS proteinopathies (153), or (iii) insert secretion signals allowing VHH to engage extracellular targets (which is desirable to prevent prion-type transmission), or allow so-called “cross-correction” in which VHH can be internalized into non-transduced cells to exert a therapeutic effect (152, 154, 155).

To date, the use of VHH in the clinics has not raised significant safety concerns (156). Importantly, VHH safety can be further enhanced through the process of humanization (157), and this increases their attractiveness for clinical applications (158). Indeed, Caplacizumab-yhdp (Cablivi^®^), a 28-kDa bivalent nanobody indicated for the treatment of thrombotic thrombocytopenic purpura (TTP), was recently granted EMA and FDA approval. Although the potential of using rAAV vectors for the long-term delivery of VHH into the CNS has yet to be fully explored, Marino et al. recently provided significant proof of concept for the feasibility of A-MAD strategies, based on VHH delivery, to stop or slow neurodegenerative disease progression, as shown by the disease modifying effects observed in a murine model of AD, following systemic administration of AAV-PHP.B encoding a highly-specific anti-BACE1 VHH (152).

In summary, it has been speculated for years that expression of VHH *in vivo*, with the aid of gene therapy vectors, could ultimately be their most powerful clinical application (159, 160). However, to take full advantage of A-MAD it will be necessary to optimize the transgene cassette to obtain robust and controllable expression of VHH in the CNS.

### Enhancing VHH Expression

The use of strategies to maximize production of biologics within the desired cell-type is beneficial for two main reasons: i) systemic administration of lower vector doses would be sufficient to reach a therapeutic concentration of the biologics within the CNS, effectively minimizing the risks of potential side effects, as well as therapy costs; ii) targeted transduction of a limited number of cells could still suffice to achieve a disease modifying effect, particularly for biologics engineered for secretion, which could subsequently be taken up by surrounding cells.

**Figure 6:**
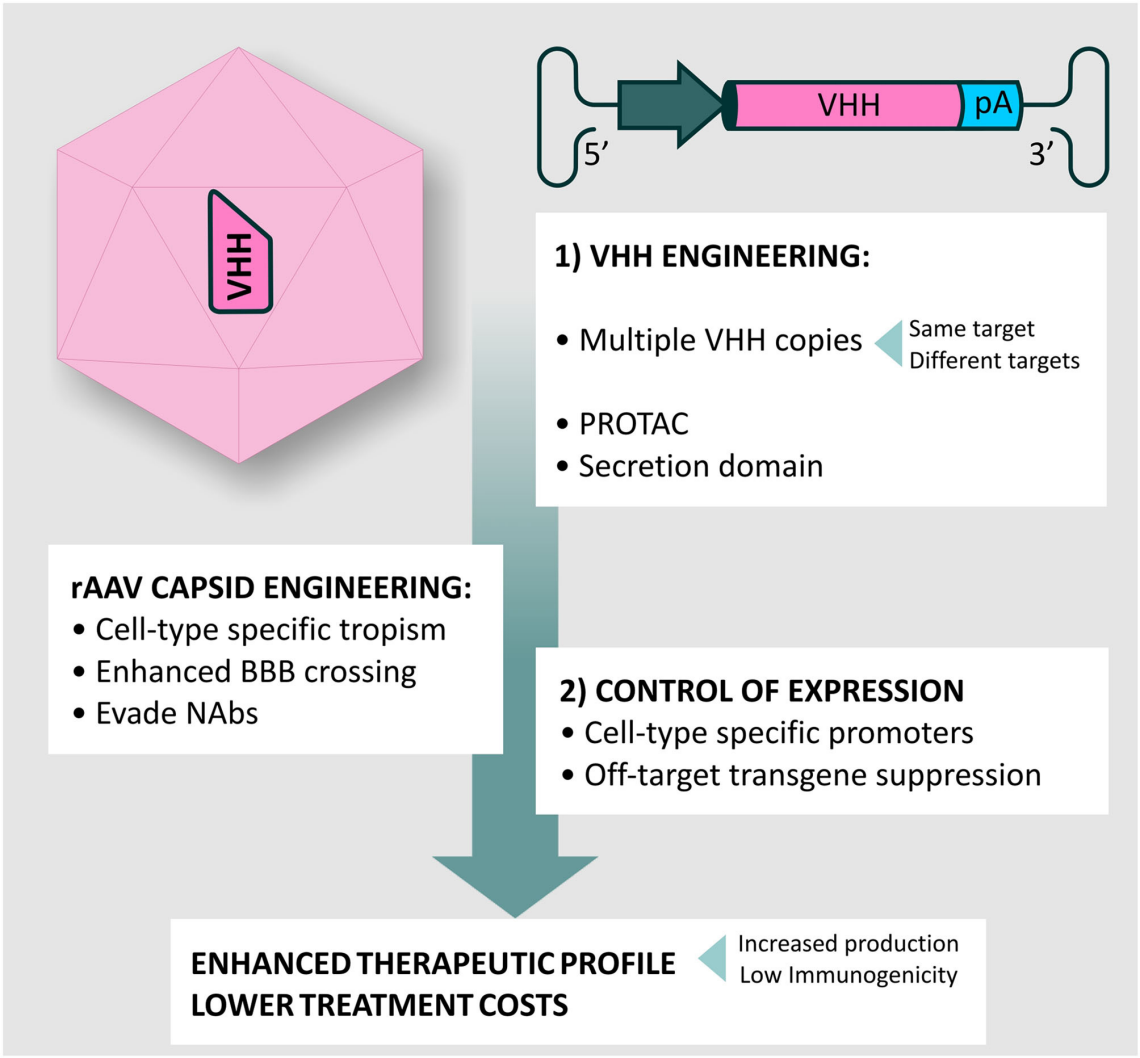
rAAV vectors and VHH engineering for A-MAD. Summary of potential modifications for A-MAD. Engineering the rAAV capsid and transgene cassette can maximize BBB crossing, NAbs evasion and spatially restrict VHH expression to the desired cell-type. More efficient production systems will lower production costs. The outstanding versatility of VHH allows easy engineering to generate multivalent drugs, or drive proteosomal degradation of toxic protein conformations via construction of Proteolysis targeting chimeras (PROTACs). Additionally, incorporation of secretion signals into the VHH will allow targeting of extracellular aggregates, preventing prion-type transmission. Together, the impact of improved rAAV vector technology and VHH engineering will produce more efficient A-MAD-based therapeutics, with concomitant reductions in undesired side-effects and lower treatment costs.

Incorporation of the WPRE element within the rAAV transgene cassette is now a routine modification, which has been shown to enhance levels of transgene expression in the murine brain and in human retina (161, 162). An exciting recent development adopted to increase transgene expression in the periphery is the use of a core promoter in combination with a so-called *cis*-acting regulatory module (CRM). These short sequences comprise clusters of evolutionary conserved transcription factor binding sites that boost transcription in a cell-type specific manner (163, 164). To date, *in silico* approaches proved successful in discovering liver-(163), cardiac-(164) and skeletal muscle- (165) specific CRMs that, upon incorporation into the transgene cassette, enhanced transgene expression up to ~100-fold in comparison with the levels achieved by core promoters alone. The identification of CRMs for CNS would represent a large step forward as mAbs levels produced by A-MAD are likely critical for the successful treatment of proteinopathies (Figure 6). Nevertheless, although VHH show low immunogenicity, the possibility of adverse immune responses to robust VHH production cannot be overlooked in the clinic. Hence, systems to spatially and temporarily regulate VHH production with the CNS are desirable.

### Promoters to Drive Transgene Expression in a Spatially Defined Manner

VHH expression can be driven within the cell-type of interest using specific promoters, overcoming issues such as (potential) toxicity resulting from off-target, multi-organ transgene overexpression, or deleterious immune responses resulting from the (undesired) transduction of antigen presenting cells (APCs) (166, 167).

Given the limited packaging capacity of rAAV vectors, short promoter sequences are beneficial, and the human synapsin 1 promoter [480 base pairs (bp) (168)] and truncated versions of the glial fibrillary acidic protein (GFAP) promoter [such as GfaABC_1_D, 694 bp (169)] have been well-characterized and are routinely used to restrict transgene expression to neurons and astrocytes, respectively (168–170). Far less characterized are short oligodendrocyte-specific promoters (171), albeit oligodendrocyte pathology seems to contribute significantly to proteinopathies, such as AD (172) and PD (173). Thus, we believe that increasing research efforts in this area should be prioritized to ensure that the full range of components driving CNS proteinopathies is effectively targeted. In this direction, ongoing research efforts are now aimed at refining patterns of transgene expression to target specific cell subtypes: for example, the mGAD65 promoter drives expression in GABAergic interneurons throughout the adult mouse brain, unlike the Dlx promoter, the previous gold standard for GABAergic interneuron targeting in the cortex, which shows mainly forebrain restricted expression (174). In addition, the mGAD65 promoter seems to preferentially drive expression in parvalbumin-positive interneurons and chandelier cells. Targeting (subsets of) GABAergic interneurons could provide significant therapeutic benefit in the treatment of tauopathies, where pathological tau accumulation has been implicated in memory deficits due to impaired GABAergic transmission (152, 175, 176).

Although cell-specific promoters significantly restrict VHH expression to the target cell type, a residual amount of off-target transgene expression cannot be excluded following systemic vector administration. To prevent undesirable effects due to “leaky” transgene expression, vector targeting can be further improved at the post-transcriptional level, including the use of micro-RNA (miRNA)-based methods to suppress transgene expression (177). As an example, Xie et al. exploited a multi-tissue miRNA-dependent suppression mechanism to spatially limit transgene expression to the CNS of adult mice following systemic AAV9 administration (178).

### Temporal Control of Transgene Expression

Even though VHH display low immunogenicity, it is not possible to foresee whether permanent VHH expression in post-mitotic CNS cells, which display little or no turnover/renewal, could potentially increase the risk of immune responses, leading to irreversible cell damage/death. Hence, in our opinion, inclusion of a safety switch would be ideal to limit adverse reactions. One interesting approach to regulating transgene expression has been to take advantage of natural variations in promoter activity: upregulation of endogenous GFAP is a well-documented occurrence in several CNS pathologies in higher vertebrates (179), and proof of concept experiments with a lentiviral vector-based system for gene delivery suggest that GFAP promoters could even be used to enhance production of a therapeutic transgene in the event of significant astrogliosis (180). However, this approach is limited by a number of key issues, including that the pattern of GFAP expression across CNS areas is highly variable, and therefore, the response to injury is highly heterogeneous (181, 182).

In contrast, the use of small molecule inducible promoter systems appears a valid option, particularly as transient therapeutic expression may still produce a therapeutic effect, as previous studies on BACE1 suggest that transient inhibition of the enzyme is sufficient to produce sustained long-term reductions of amyloid-β (Aβ) accumulation in AD (183, 184). An ideal inducible system should be completely free of endogenous influence, and would be built around: (i) an inducible promoter dependent on a unique regulatory DNA sequence exogenous to the host genome; (ii) an effector protein that recognizes and binds specifically to this regulatory sequence, without interfering with any other sites within the host genome and iii) dose-dependent inducibility of the effector protein by a BBB penetrant drug that can be safely and repeatedly administered. Although reports of such systems in the literature are limited to date, the inducer/repressor tetracycline (Tet)-dependent system is an interesting example, and was successfully employed to modulate transgene expression for up to 5 years in the muscle of non-human primates, following locoregional intravenous AAV administration (185). However, delayed humoral and cellular immune responses directed against the bacterial component of the Tet-dependent system have been reported in some non-human primate studies (186, 187). Although these issues appear to have been overcome by fusing the reverse tetracycline-controlled transactivator (rtTA) with a glycine-alanine repeat (GARrtTA), which is known to enable the Epstein-Barr virus to evade host immune response (188, 189), caution is obviously needed moving forward. In the context of the CNS, the rtTA effector minocycline (190) appears particularly suited for the inducible expression of biologics, as it shows (i) high BBB permeability, (ii) low cytotoxicity [albeit minor side effects can be observed upon prolonged administration (191, 192)] and (iii) neuroprotective (anti-apoptotic and anti-inflammatory) effects, dependent on the inhibition of cytosolic cytochrome C translocation (193), caspase activity (194), microglial proliferation (195), and activation of inducible nitric oxide synthase and cytokine release in PD, HD, ALS, and cerebral ischemia (196, 197). These properties suggest minocycline-controlled systems (or equivalents) are likely to play an important role in the development of rAAV-mediated strategies (including A-MAD) for CNS use.

## Discussion

In the last four decades, the use of mAbs as therapeutics has rapidly expanded, with many now “blockbuster drugs,” which generate billions of dollars of annual revenue. As of November 2020, 88 antibody therapeutics were in stage 3 clinical studies for a variety of conditions (other than COVID-19) (198), and when combined with a relatively fast approval rate (currently twice as fast as for small molecules) their dominant position in the market place is likely to continue (198). In general, however, the approval of mAbs for CNS-related indications is lagging behind, albeit with a few noteworthy exceptions (50, 199–204). This is not surprising, as perhaps the biggest challenge in the development of non-invasive strategies to target CNS disorders is overcoming the restricted permeability of the BBB, which significantly limits mAbs access to the CNS from the systemic circulation. rAAV vector technology offers an interesting solution to this problem, with novel BBB-crossing serotypes allowing extended production of biologics within the CNS following a single systemic injection.

Interestingly, gene therapy-based methods are becoming increasingly popular for the treatment of previously incurable diseases. In fact, in 2019, the FDA authored a report predicting that by 2025 ~10–20 novel cell and gene therapy products will be approved per year (205). A comprehensive analysis of the 94 AAV-based clinical trials completed to date shows that the focus is directed towards four main therapeutic areas: liver, muscle, retinal and CNS diseases. The general trend towards optimism is in no small part due to the fact that in the fields of neurology and ophthalmology, areas with traditionally high rates of clinical trial failures, ~30% of the tested rAAV vector-based drugs successfully completed the path from investigational new drug (IND) to new drug application (NDA), a percentage exceeding the historical averages of any other drug type (206). The potential of AAVs as drug delivery platforms, particularly if supported by further successful outcomes in clinical trials, will undoubtedly drive research aimed at further improving and refining the technology, particularly for use in areas such as the CNS, where conventional small molecule therapies have traditionally had long developmental times and high failure rates (136). In fact, recent progress in both capsid engineering and manufacturing technology suggest that widespread gene delivery to the adult CNS via intravenous administration is now possible, while improvements in antibody engineering and expression cassette design point towards safe and effective A-MAD-based strategies for chronic, multi-region CNS diseases.

Given their unique properties, which are ideally suited for A-MAD, we anticipate use of VHH will play a key role in developing the technology, particularly as further research provides greater insights into disease causing mechanisms and identifies future targets for therapeutic intervention. The push towards using VHH in CNS disease is exemplified by the current efforts of major Biotech and Pharma companies, such as Ablynx (Sanofi) and Boehringer Ingelheim, who partnered to explore a potential VHH-based treatment for Alzheimer's disease; as of early 2022, the result of this partnership is an ongoing phase 1 clinical trial in AD patients for the Aβ-targeting biparatopic nanobody, NbBI.1031020 (207).

Nevertheless, some open concerns remain to be addressed before the clinical adoption of A-MAD in earnest. First and foremost, reports that the enhanced BBB crossing of AAV-PHP.B is restricted to BL6 mice and absent in non-human primates should be considered a cautionary tale in the rush to develop new AAV-based delivery platforms. In future, time and effort need to be invested in developing screening platforms which avoid issues arising out of cross-species differences in BBB composition and immune responses. Luckily, the growing opportunities to test vector performance in 3D cerebral organoids (208–210), which recapitulate aspects of the human brain, offer major advantages for the development of CNS relevant rAAV vectors. Second, and an issue specific to the use of VHH technology, is whether the lack of an Fc region is beneficial, reducing the risk of neuroinflammation and edema (211), or whether the effector function is actually needed. In all probability, this is likely dependent on the mechanism of VHH action; for example, inhibition of BACE1 activity in AD does not require an effector domain (152). In the event that effector function is required, however, the ease with which VHH can be engineered allows the straight-forward incorporation of the Fc region from human IgG, thus generating bivalency and effector function (212). Third, in the majority of cases, disease onset and the accumulation of irreversible cellular damage is thought to precede the appearance of symptoms by many years (213–216), raising the issue of when treatment should be initiated. Hence, it is likely that improvements in therapeutics and delivery systems will need to be mirrored by improvements in biomarker detection and cognitive testing.

Finally, methods for efficient, high-quality, large-scale manufacture of AAV vectors, exploiting easily scalable production systems [baculovirus–Sf9-systems (217); recombinant HSV-based systems (218); adenovirus–HeLa cell systems (219)] will ease the translation of A-MAD into clinical use, by significantly cutting production costs, which remain high at present. As an example, in early 2022, the cost of Zolgensma, indicated for children aged <2 years, is $2.48 million for a single treatment, and in all likelihood the higher doses required for adult therapeutics will be reflected in even higher costs. Nevertheless, it should be pointed out that A-MAD strategies would rely on a “single-dose” therapeutic approach, due to the ability of rAAV to effectively transform specific CNS cell-types into “biopharmacies,” capable of long-term biologic production. In contrast, high level dosing of standard, non-vector-based mAbs therapies, repeated over the course of decades, would itself be extremely expensive, allowing us to speculate that the final costs will in the end be potentially comparable.

To conclude, the growing toolbox available for A-MAD supports a bright future in which the full range of CNS proteinopathies can be effectively treated. Combining the cutting-edge power of rAAV vectors for CNS targeting with the unique properties of VHH promises to revolutionize our approach to CNS disease: the future is now!

## Author Contributions

Both authors listed have made a substantial, direct, and intellectual contribution to the work and approved it for publication.

## Funding

MM was supported by a pre-doctoral fellowship from the Fonds Wetenschappelijk Onderzoek (FWO) (SB/1S48018N). Work in the Holt lab has been supported by the Thierry Latran Foundation (SOD-VIP), FWO (Grant 1513616N), and European Research Council (ERC) (Starting Grant 281961 – AstroFunc; Proof of Concept Grant 713755 – AD-VIP). MGH is currently the ERANet Chair (NCBio) at i3S Porto funded by the European Commission (H2020-WIDESPREAD-2018-2020-6; NCBio; 951923).

## Conflict of Interest

The authors declare that the research was conducted in the absence of any commercial or financial relationships that could be construed as a potential conflict of interest.

## Publisher's Note

All claims expressed in this article are solely those of the authors and do not necessarily represent those of their affiliated organizations, or those of the publisher, the editors and the reviewers. Any product that may be evaluated in this article, or claim that may be made by its manufacturer, is not guaranteed or endorsed by the publisher.
